# Supplementary material for: From sequence to enzyme mechanism using multi-label machine learning
Source: BMC Bioinformatics. 2014 May 19;15:150. doi: 10.1186/1471-2105-15-150 (PMC4229970; doi:10.1186/1471-2105-15-150)
Supplement: Additional file 2 — Java code of ml2db. Additional file ml2db_code.tar.gz contains the Java source code to run the multi-label machine learning experiments and save the results to database. The code’s Javadoc is included. [file 1471-2105-15-150-S2.zip › additional file 2/ml2db/ml2db/doc/mulan/dimensionalityReduction/BinaryRelevanceAttributeEvaluator.Rank.html]

BinaryRelevanceAttributeEvaluator.Rank


JavaScript is disabled on your browser.


- Overview
- Package
- Class
- Use
- Tree
- Deprecated
- Index
- Help

- Prev Class
- Next Class

- Frames
- No Frames

- All Classes

- Summary:
- Nested |
- Field |
- Constr |
- Method

- Detail:
- Field |
- Constr |
- Method


mulan.dimensionalityReduction

## Class BinaryRelevanceAttributeEvaluator.Rank

- java.lang.Object
- - mulan.dimensionalityReduction.BinaryRelevanceAttributeEvaluator.Rank

- All Implemented Interfaces:
  :   java.lang.Comparable

  Enclosing class:
  :   BinaryRelevanceAttributeEvaluator

  ---

    

  ```
  public class BinaryRelevanceAttributeEvaluator.Rank
  extends java.lang.Object
  implements java.lang.Comparable
  ```

  a wrapper class for score-based attribute ranking

- - ### Constructor Summary

    Constructors

    Constructor and Description

    `BinaryRelevanceAttributeEvaluator.Rank(double score,
    int index)`

    constructor
  - ### Method Summary

    Methods

    | Modifier and Type | Method and Description |
    | --- | --- |
    | `int` | `compareTo(java.lang.Object o)` |
    | `int` | `getIndex()` Returns the index of the attribute |
    | `double` | `getScore()` Returns the score of the attribute |

    - ### Methods inherited from class java.lang.Object

      `equals, getClass, hashCode, notify, notifyAll, toString, wait, wait, wait`

- - ### Constructor Detail


    - #### BinaryRelevanceAttributeEvaluator.Rank

      ```
      public BinaryRelevanceAttributeEvaluator.Rank(double score,
                                            int index)
      ```

      constructor

      Parameters:
      :   `score` - the score to be given
      :   `index` - the index to be given
  - ### Method Detail


    - #### getScore

      ```
      public double getScore()
      ```

      Returns the score of the attribute

      Returns:
      :   score of the attribute


    - #### getIndex

      ```
      public int getIndex()
      ```

      Returns the index of the attribute

      Returns:
      :   index of the attribute


    - #### compareTo

      ```
      public int compareTo(java.lang.Object o)
      ```

      **Specified by:**
      :   `compareTo` in interface `java.lang.Comparable`


- Overview
- Package
- Class
- Use
- Tree
- Deprecated
- Index
- Help

- Prev Class
- Next Class

- Frames
- No Frames

- All Classes

- Summary:
- Nested |
- Field |
- Constr |
- Method

- Detail:
- Field |
- Constr |
- Method
